# A Real-World Data Driven Pharmacovigilance Investigation on Drug-Induced Arrhythmia Using KAERS DB, a Korean Nationwide Adverse Drug Reporting System

**DOI:** 10.3390/ph16111612

**Published:** 2023-11-15

**Authors:** Chaerin Go, Semi Kim, Yujin Kim, Yongjun Sunwoo, Sae Hyun Eom, Jiseong Yun, Sooyoung Shin, Yeo Jin Choi

**Affiliations:** 1 Department of Pharmacy, College of Pharmacy, Kyung Hee University, Seoul 02447, Republic of Korea; 2 Department of Regulatory Science, Graduate School, Kyung Hee University, Seoul 02447, Republic of Korea; 3 Department of Pharmacy, College of Pharmacy, Ajou University, Suwon 16499, Republic of Korea; 4 Research Institute of Pharmaceutical Science and Technology (RIPST), Ajou University, Suwon 16499, Republic of Korea; 5 Institute of Regulatory Innovation through Science (IRIS), Kyung Hee University, Seoul 02447, Republic of Korea

**Keywords:** adverse drug events, arrhythmia, drug safety, KAERS DB, pharmacovigilance, RWD

## Abstract

This study aims to investigate the prevalence and seriousness of drug-induced arrhythmia and to identify predictors associated with the seriousness of arrhythmia. Drug-induced arrhythmia cases reported to the Korean Adverse Event Reporting System Database (KAERS DB) from January 2012 to December 2021 were investigated. A disproportionality test was performed to detect the association of the etiologic medication classes and types, along with patient demographic information, with the seriousness of drug-induced arrhythmia. Logistic regression was performed to investigate the predictors that increase the risk of serious arrhythmia. The most common etiologic agent for drug-induced arrhythmia was sevoflurane, whereas serious arrhythmia was most prevalent with narcotics. Antibiotics (reporting odds ratio (ROR) 4.125; 95% CI 1.438–11.835), chemotherapy (ROR 6.994; 95% CI 2.239–21.542), and iodinated contrast media (ROR 8.273; 95% CI 3.062–22.352) had a strong association with the seriousness of drug-induced arrhythmia. Among numerous etiologic agents, ioversol (ROR 16.490; 95% CI 3.589–75.772) and lidocaine (ROR 12.347; 95% CI 2.996–50.884) were more likely to be reported with serious arrhythmia. Aging and comorbidity, primarily cancer, are the most contributing predictors associated with serious arrhythmia. Further studies on the clinical significance of patient-specific predictors for the increased risk of serious drug-induced arrhythmia are warranted to promote drug safety.

## 1. Introduction

Arrhythmia refers to any abnormal cardiac condition involving an aberrant rate and rhythm induced by abnormal electrical stimulation and remains a critical global health issue [1,2]. The prevalence of arrhythmia increases each year, and studies suggest that the prevalence of atrial fibrillation, the most common type of arrhythmia, has increased by at least three-fold over the last 50 years, responsible for 46.3 million patients in 2016. Considering the broad classifications of arrhythmia, including bradycardia, tachycardia, supraventricular, and ventricular arrhythmias, the overall prevalence may be substantially greater than the estimation [3,4]. Arrhythmia has also been nominated as the major cause of sudden cardiac arrest and sudden cardiac death, accounting for 276,373 cardiovascular deaths from January 2011 to December 2018 [3,5], and the new-onset atrial fibrillation markedly increases the risk of ventricular arrhythmias involving ventricular tachycardia and ventricular fibrillation, eventually provoking sudden cardiac death in many patients [6]. Additionally, arrhythmia substantially elevates the risk for heart failure, stroke, and dementia. Considering that the majority of patients with arrhythmia already have additional comorbidities, including major cardiovascular disease, diabetes mellitus, and respiratory disorders, patients with arrhythmia are anticipated to have a greater risk of morbidity and mortality, subsequently decreasing quality of life during the course of illnesses [5,7].

Arrhythmia is triggered by various factors, including comorbidities and modifiable lifestyle elements, and drugs can also be potential triggers for arrhythmia, as seen in cases where medications reported with cardiac arrhythmia as adverse events (AEs) have been withdrawn from the market [3,8,9,10]. Interestingly, among numerous types of medications with high arrhythmic potentials, anti-arrhythmic agents such as adenosine phosphate, encainide, and tocainide were frequently withdrawn from the market between 1950 and 2017 [10]. Nonetheless, drug-induced arrhythmia is often underestimated despite considerable adverse clinical outcomes due to the low reported incidence [11,12,13], as implied by the incidence of 2.5 and 4.0 per 1,000,000 person years in men and women, respectively [13]. Moreover, in many cases, mortality induced by drug-induced arrhythmia is often underreported in the real world, as the majority of patients who die of sudden cardiac death already possess multiple severe comorbidities that could have also contributed to sudden cardiac death, thereby further contributing to the underestimation of the incidence and the seriousness of drug-induced arrhythmia [12]. Patients with an increased risk of drug-induced arrhythmia usually have multiple risk factors, including prolonged QT intervals, cardiovascular diseases such as acute myocardial infarction (MI) and heart failure, and electrolyte disorders such as hypokalemia, hypomagnesemia, and hypocalcemia. Aging indeed further increases the susceptibility to drug-induced arrhythmia, consequently augmenting the risk of adverse clinical outcomes, including hospitalization and death [11,13]. Despite these challenges, the optimal guidelines aimed at preventing drug-induced arrhythmia are still limited, implying imperative needs for pharmacovigilance investigations on drug-induced arrhythmia to endorse drug safety [13].

While Asians typically experience a lower incidence of arrhythmia than other ethnicities, arrhythmia remains a critical health problem in Korea as well, as implied by the constantly increasing number of newly diagnosed arrhythmia patients in recent years [3,14]. According to the statistics from the Health Insurance Review and Assessment Service, the number of arrhythmia patients skyrocketed by 22.1% between 2016 and 2020, accounting for 400,682 patients in 2020 [15], and the prevalence of arrhythmia is expected to increase much faster than the estimation as the number of aging populations accelerates. Nevertheless, little is known about the prevalence, risk factors, and common etiologic agents related to drug-induced arrhythmia in Korean populations. The American Heart Association has identified culprit medications that pose a high risk of inducing arrhythmia, such as antiarrhythmics, anesthetics, antidepressants, antihypertensives, antipsychotics, immunotherapy, and opioids; however, the clinical significance of these agents in the Korean population remains uncertain [13]. Previous studies have indicated variations in the risk of drug-induced arrhythmia across diverse ethnicities; Caucasians, for instance, possess a relatively larger number of genetic traits that make them more susceptible to drug-related arrhythmia than other ethnicities [3,16]. However, this does not mean that the incidence of drug-induced arrhythmia in the Asian population should be neglected. Hence, this study aimed to assess the prevalence and seriousness of drug-induced arrhythmia, identify the culprit agents associated with drug-induced arrhythmia, and explore predictors that may contribute to an increased risk of serious arrhythmia, utilizing real-world data (RWD) obtained from the Korean Institute of Drug Safety and Risk Management-Korean Adverse Event Reporting System database (KAERS DB), a nationwide voluntary adverse drug event (ADE) reporting system, to convey evidence on the safety of medication associated with a high risk of arrhythmia in Korea.

## 2. Results

### 2.1. Baseline Demographics

**Figure 1:**
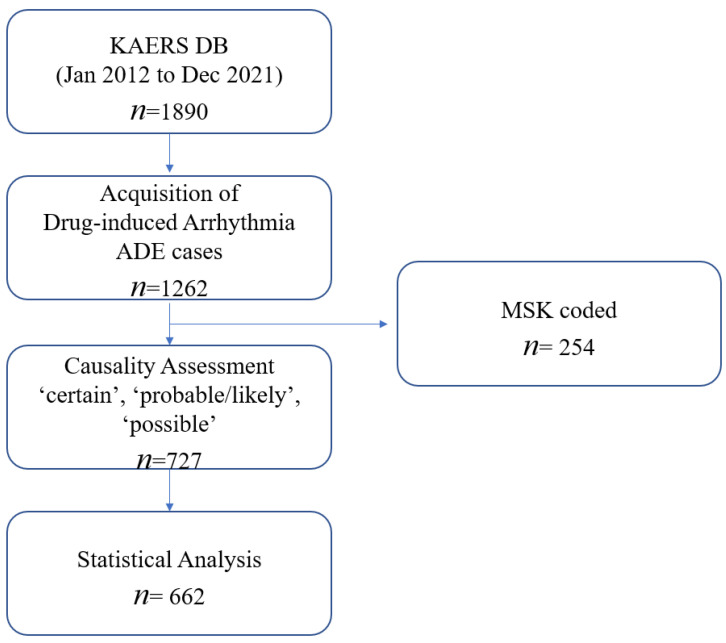
Data acquisition process of the study.

Among 1890 cases extracted from KAERS DB, a total of 662 ADE cases were eligible for analysis, in accordance with the World Health Organization—Uppsala Monitoring Center (WHO-UMC) causality assessment from January 2012 to December 2021 (Figure 1). The demographic information of the included ADE cases is summarized in Table 1. The majority of drug-induced arrhythmia cases were reported by the doctors (*n* = 655; 98.9%). Men reported more drug-induced arrhythmia (*n* = 364; 55.0%) than women (*n* = 292; 44.1%). As for age, those aged between 60 and 79 were reported to have the most ADE cases (*n* = 197; 29.8%). The most common indication or comorbidity associated with drug-induced arrhythmia ADEs was anesthesia (*n* = 328; 49.5%), followed by cancer (*n* = 57; 8.6%), cardiovascular disorders (*n* = 55; 8.3%), and infections (*n* = 47; 7.1%). Only 87 patients (13.2%) reported at least two concurrent medications, whereas the majority of patients reported one medication use (*n* = 575; 86.9%).

The most common indication or comorbidity associated with drug-induced arrhythmia ADEs was anesthesia (*n* = 328; 49.5%), followed by cancer (*n* = 57; 8.6%), cardiovascular disorders (*n* = 55; 8.3%), and infections (*n* = 47; 7.1%). The most common etiologic agent for drug-induced arrhythmia was sevoflurane (*n* = 328; 49.55%), followed by propacetamol hydrochloride (*n* = 22; 3.32%) and pethidine hydrochloride (*n* = 18; 2.72%) (Table 2). The prevalence of SAE related to drug-induced arrhythmia was 8% (*n* = 53), mostly reported with life-threatening conditions (*n* = 20; 37.7%) and hospitalization (*n* = 19; 35.8%). Among 53 cases of SAEs, 9 cases (16.98%) were induced by narcotic analgesics, including fentanyl citrates (*n* = 6; 11.32%) and pethidine hydrochloride (*n* = 3; 5.66%), and 7 cases (13.21%) were induced by contrast media, primarily iohexol (*n* = 3; 5.66%) and ioversol (*n* = 4; 7.55%).

### 2.2. Association between Seriousness of Drug-Induced Arrhythmia and Medication Classes

**Figure 2:**
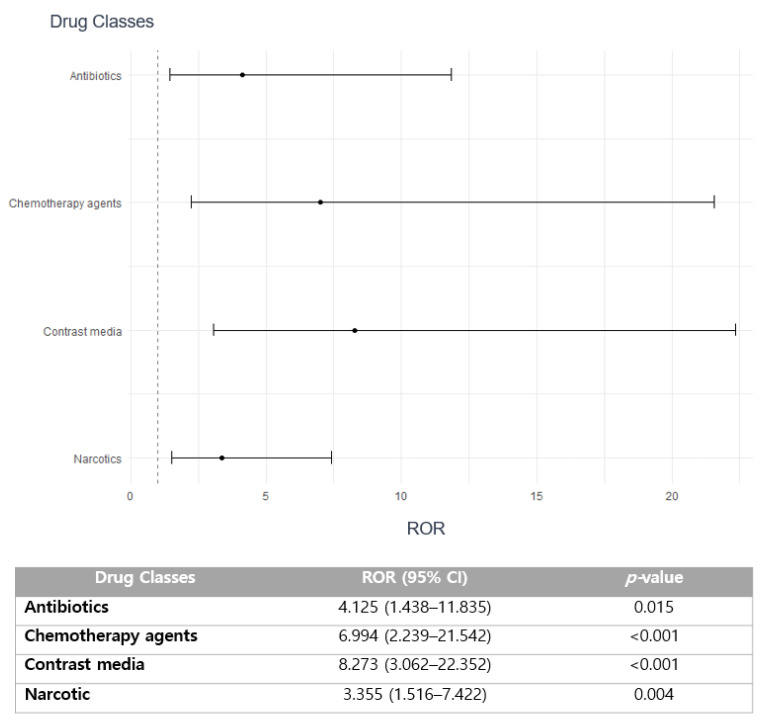
Association of medication classes with the seriousness of drug-induced arrhythmia.

The substantial association with seriousness of ADEs was noted in antibiotics (reporting odds ratio (ROR) 4.125; 95% confidence interval (CI) 1.438–11.835; *p =* 0.015), chemotherapy (ROR 6.994; 95% CI 2.239–21.542; *p <* 0.001), contrast media (ROR 8.273; 95% CI 3.062–22.352; *p <* 0.001), and narcotic agents (ROR 3.355; 95% CI 1.516–7.422; *p <* 0.001) (Figure 2).

### 2.3. Association between Seriousness of Drug-Induced Arrhythmia and Medications

**Figure 3:**
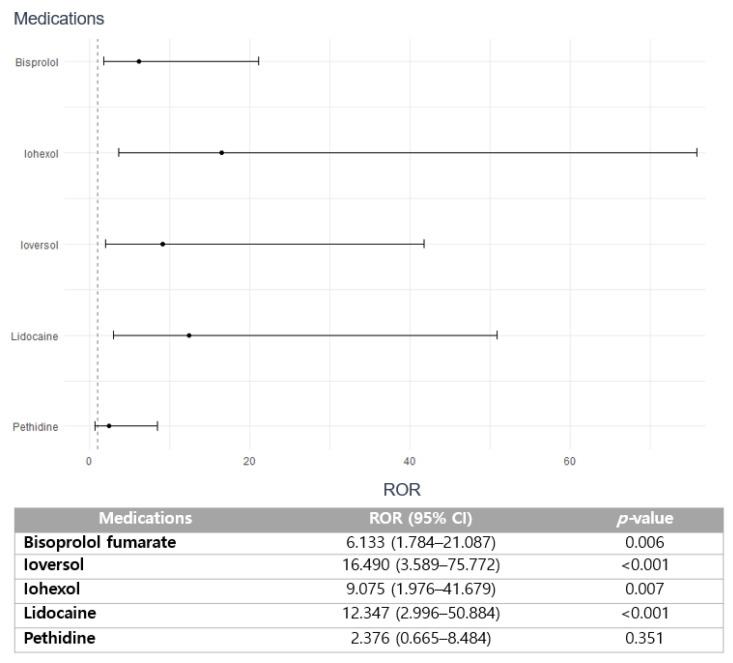
Association of medications with the seriousness of drug-induced arrhythmia.

Medications including bisoprolol fumarate (6.133; 95% CI 1.784–21.087; *p =* 0.006), ioversol (ROR 16.490; 95% CI 3.589–75.772; *p <* 0.001), iohexol (ROR 9.075; 1.976–41.679; *p =* 0.007), and lidocaine (ROR 12.347; 95% CI 2.996–50.884; *p <* 0.001) are strongly correlated with the increased risk of reporting SAEs (Figure 3). However, no statistical significance on the association between seriousness of arrhythmia and pethidine is observed (ROR 2.376; 95% CI 0.665–8.484; *p* = 0.354).

### 2.4. Predictors Associated with an Increased Risk of Serious Arrhythmia

Increasing age has a significant association with an increased risk of reporting serious drug-induced arrhythmia (ROR 5.153; 95% CI 1.984–13.379), whereas sex has a statistically insignificant association with an increased risk of SAEs (Table 3). Concomitant medication administration with etiologic medication was not associated with a risk of reporting serious drug-induced arrhythmia. The presence of comorbidity, otherwise referred to as patient history, may increase the risk of reporting serious arrhythmia (ROR 2.089; 95% CI 1.000–4.367), and a history of cancer may be associated with a significantly increased risk of serious arrhythmia ADEs (ROR 2.779; 95% CI 1.008–7.666). However, multiple logistic regression revealed a statistically insignificant contribution of these patient-specific clinical predictors to the elevated risk of SAEs. Nonetheless, men have a slightly higher risk of developing serious drug-induced arrhythmia than women [ROR 1.480 (95% CI 0.826–2.654); odds ratio (OR) 1.698 (95% CI 0.884–3.263)].

## 3. Discussion

Most drug-induced arrhythmia cases were reported with anesthetic agents, mostly sevoflurane, followed by antipyretic anti-inflammatory drugs and narcotic analgesics (Table 2). The incidence of serious drug-induced arrhythmia was 8% of all studied drugs, primarily manifested as hospitalizations or life-threatening events (Table 1). The most common culprit class for SAEs related to drug-induced arrhythmia was narcotic analgesics, including fentanyl and pethidine (Table 2). The majority of ADE cases were reported by doctors, followed by pharmacists and nurses (Table 1). Disproportionality analysis revealed that drug classes involving antibiotics, chemotherapeutic agents, iodinated contrast media, and narcotic analgesics are more likely to be associated with serious drug-induced arrhythmia (Figure 2). Furthermore, aging, in addition to the presence of a medical history, primarily cancer, are the essential predictors that have a strong correlation with an increased risk of reporting serious drug-induced arrhythmia (Table 3).

Torsade de Pointes (TdP), manifested as QT prolongation and polymorphic ventricular tachycardia, stands as the primary cause of drug withdrawals from the market [17,18], and anti-arrhythmic medications such as disopyramide, procainamide, quinidine, and sotalol are reported to have the most cases of drug-induced arrhythmia, including TdP [17,19]. However, in this study, no anti-arrhythmic agents were reported for drug-induced arrhythmia. Instead, anesthetics including sevoflurane, propofol, and lidocaine were the most common etiologic agents for drug-induced arrhythmia (Table 2). This study revealed similar findings with the American Heart Association: sevoflurane and propofol are two major anesthetics with high arrhythmic potentials, including TdP [13,18,20,21]. Interestingly, sevoflurane-induced ADE cases accounted for almost 50% of all drug-induced arrhythmia ADE reports, though nonserious (Table 2). Sevoflurane has been commonly prescribed due to its favorable profiles for airway management and hemodynamic stability [22]. However, a recent retrospective analysis addressed the potential risk of arrhythmia associated with sevoflurane in Korea; approximately 17.3% of patients developed arrhythmia, mainly presented as tachycardia, after anesthetic induction with sevoflurane, and administration of maximal concentration from the beginning of induction was the strong risk factor for the increased risk of arrhythmia, implying that sevoflurane-induced arrhythmia can be preventable with safe medication use [22]. Nonetheless, guidelines on optimal anesthesia induction with sevoflurane are currently limited in Korea, despite its prevalent use. Hence, further studies investigating the risk of arrhythmia by anesthetic agents, including sevoflurane, in consideration of confounding factors such as preexisting electrocardiographic (ECG) abnormalities as well as medications concurrently administered are warranted for the safe use of anesthetics [23].

Opioids have diverse SAE profiles, including sedation, hypotension, dependency, and tolerance [24,25], and this study displayed a substantially high risk of serious arrhythmia with narcotic agents, primarily fentanyl and pethidine (Table 2). Opioids have been reported to have a high risk of arrhythmia manifested as QT prolongation and TdP, and the American Heart Association identified morphine as the etiologic agent that causes or exacerbates atrial fibrillation [13]. Although opioids are associated with hypotension and bradycardia in many patients, consequently leading to arrhythmia, underlying comorbidity may be a confounding factor for opioid-induced arrhythmia, and disproportionality analysis revealed that the presence of a medical history, especially cancer, increases the likelihood of reporting serious arrhythmia (Table 3). In general, opioids are commonly prescribed to cancer patients who have altered pharmacokinetic and pharmacodynamic profiles secondary to disease progression, and these patients concomitantly administer numerous medications, including those with high arrhythmic potentials, such as chemotherapeutic agents [13,26]. Moreover, these patients are prone to infections due to numerous cycles of chemotherapy, subsequently having increased exposures to antibiotics and antifungals, which are also considered potential inducers of arrhythmia [13,27]. However, the most ADE cases with opioid-induced arrhythmia in this study were reported in patients with noncancer pain, and the culprit agent for serious arrhythmia was fentanyl, which has minimal effects on the cardiovascular system, including QT prolongation, compared to other opioids [28]. Although the evident mechanism for the incidence of fentanyl-induced arrhythmia in this study is not available at this time, comorbidity and concurrent use of other medications with fentanyl may have raised the incidence of serious drug-induced arrhythmia as it is commonly used as sedation for diverse medical procedures [29].

The distinctive finding of this study is the strong association between iodinated contrasts, including ioversol and iohexol, and the severity of drug-induced arrhythmia (Figure 3). This finding aligns with recent case reports suggesting a potentially elevated risk of atrial fibrillation after intravenous administration of iodinated contrasts [30]. The most common clinical manifestation of iodinated contrast-induced AEs is dermatologic reactions, including pruritis and urticaria, and acute kidney injury or nephropathy, which indeed provide significant adverse complications in patients, leading to high morbidity and mortality [31,32]. Nonetheless, the evident correlation between cardiac manifestations associated with these agents is yet to be determined. Currently, some studies suggest that the immediate cardiac findings such as cardiovascular arrhythmia, cardiovascular shock, and cardiac arrest after administration of iodinated contrasts may be associated with hemostatic dysfunction, a reduced volume of venous return, contrast-induced thyrotoxicosis, a direct cardiac mechanism, or a lowered threshold of ventricular contraction [31,33,34,35]. Conversely, underlying comorbidity may amplify the risk of contrast-induced arrhythmia, as suggested by the study, which demonstrated a higher incidence of contrast-induced arrhythmia in patients with acute MI or recent infarction [36]. Furthermore, drawing from a prior pharmacovigilance investigation conducted by our research group, the Korean population is more susceptible to the AEs induced by iodinated contrast media, and this may have augmented the risk of serious arrhythmia [37]. Nevertheless, treatment guidelines pertaining to the optimal use of contrast media are currently lacking. Hence, further pharmacovigilance investigations on iodinated-contrast media as well as mechanistic studies on the impact of arrhythmia induction are particularly crucial within the Korean population.

Several studies have suggested that age, women, prolonged QT intervals, and structural heart disease are essential risk factors for drug-induced arrhythmia [13,38,39]. The incidence as well as patterns of arrhythmia symptoms are strongly influenced by sex, and sex differences also affect responses to arrhythmia management, including medical therapy and catheter ablation [40,41]. Women are usually predisposed to a higher risk of drug-induced arrhythmia due to their faster resting heart rate and longer rate-corrected QT interval (QTc) than men [42,43,44]. Meanwhile, predictors associated with the seriousness of drug-induced arrhythmia are not evidently established, and this study demonstrated that increasing age is more likely to be associated with serious drug-induced arrhythmia than sex, as indicated by the results from disproportionality analysis (Table 3). Similarly, previous studies suggested age was the most compelling risk factor for cardiovascular diseases, including arrhythmia, and higher arrhythmia incidence and risk of other secondary complications induced by arrhythmia in women were closely correlated with a higher age of diagnosis and longer life expectancy than men, subsequently resulting in more adverse clinical outcomes [41]. Nonetheless, judicious monitoring of drug-induced arrhythmia when administering medications with high arrhythmic potentials is warranted regardless of sex, as both men and women with a high risk of arrhythmia typically have multiple comorbidities and numerous concurrent medications that may accentuate the arrhythmic risks. Additionally, a tentative investigation on the risk stratification of drug-induced arrhythmia is required.

The number of concomitant medications as well as medication classes may also contribute to drug-induced arrhythmia. Generally, anti-arrhythmic agents are reported in numerous cases of drug-induced arrhythmia due to their pharmacological mechanism of action (MOA); for instance, sinus bradycardia is fairly common with beta-blockers, as these agents slow down the heartbeat [45]. Nonetheless, this study may contribute to the current body of literature as we identified medications with an uncommon, rare, or unknown risk of drug-induced arrhythmia from the pharmacovigilance investigation. Moreover, although the number of ADE cases is low, psychotropic agents may have the underlying potential to increase the risk of serious arrhythmia. Hence, further investigation on the pharmacological impact on arrhythmia is highly required. On the other hand, this study did not demonstrate the significant impact of concomitant medication administration on the risk of serious drug-induced arrhythmia (Table 3), and this may be associated with under-reported information on concomitant medications. Despite substantial comorbidities such as cancer, cardiovascular disease, and diabetes mellitus, the majority of ADE cases were reported with etiologic medication, and this may contribute to the underestimation of the risk of serious arrhythmia associated with multiple drug use. Hence, promoting voluntary ADE reporting practice is required in clinical settings to endorse patient safety through pharmacovigilance activity.

Pharmacovigilance, a surveillance activity pertaining to the detection, assessment, understanding, and prevention of ADEs, is essential to promoting drug and patient safety [46]. ADEs are identified in the majority of clinical trials; however, identifying ADEs occurring in low frequency as well as ADEs related to long-term medication use is challenging in most clinical trials [47]. Thus, pharmacovigilance investigations utilizing RWD are warranted to detect infrequent ADEs in many cases, and our study identified medications that are classified as having an uncommon, rare, or unknown risk of drug-induced arrhythmia, emphasizing the benefits of RWD-derived pharmacovigilance studies (Table 2). Moreover, the benefits of RWD-based pharmacovigilance involve providing well-represented examples of real clinical settings, and considering that drug-induced arrhythmia is a more insidious ADE than many other drug-related ADEs, RWD-derived pharmacovigilance is obligated. Nevertheless, one of the largest drawbacks of RWD-based pharmacovigilance investigations involves limited data and obscure causality. Limited data usually involves under-reporting of ADE cases. In this study, although the majority of ADE records were reported by the doctors, the reporting rate was substantially low: 0.59% for doctors (655 ADE reports among 109,937 doctors), 0.01% for pharmacists (5 ADE reports among 40,388 pharmacists), and 0.001% for nurses (2 ADE reports among 240,307) when we consider the numbers of active healthcare professionals in 2021 [48]. Thus, active ADE reporting practice should be endorsed in clinical settings.

Obscure causality is another limitation involved with RWD-derived pharmacovigilance investigations. Responses to medications are usually affected by patient and disease-related factors, including pharmacokinetic/pharmacodynamic characteristics, comorbidities, drug interactions, and polypharmacy, thereby making evident decisions on ADE causality assessment difficult [49]. In this study, only 11 ADE cases, representing 1.7% of all ADE cases, were identified as having certain causality, whereas more than 500 ADE cases were classified as having “possible” causality. Furthermore, the majority of patients had comorbidities such as cardiovascular disorders, cancers, and infections, and these comorbidities not only alter medication responses but also increase medication exposures, which further induced medication-related problems, including drug interactions and inappropriate medication use. Hence, further studies on detecting safety signals related to drug-induced arrhythmia are warranted to strengthen the reliability and clinical significance of the pharmacovigilance investigation.

Despite the benefits of RWD utilization for pharmacovigilance investigations, some limitations should be acknowledged. KAERS DB is a spontaneous voluntary ADE reporting system; hence, caution is required when generalizing the study results due to substantial missing information, including comorbidity, concurrent medication therapy, and indications, as well as potential under-reporting of AEs. For instance, information on age was missing in more than 50% of ADE reports, and the number of concurrent medications is substantially lower when considering underlying medical history and indications of drug use. Additionally, as KAERS DB is a voluntary reporting system in Korea, underreporting of ADEs and study populations with a lower risk of arrhythmia may result in a biased estimation of the prevalence of drug-induced arrhythmia. Moreover, inter-reporter variability might be present during the ADE reporting step. Nonetheless, as all ADE reports included in this study were recorded by health care professionals, mostly doctors in this study, and further investigated by a review board appointed by the Korean Institute of Drug Safety and Risk Management (Ministry of Food and Drug Safety) based on medical records, interviews, and scientific data, the minimized bias and accuracy of the collected data are assured. Additionally, due to the nature of this study design and small sample sizes, especially for disproportionality analysis on the association between medications and the seriousness of arrhythmia, the reliability of the statistical significance of the analysis may be curtailed. Thus, caution should be exercised when interpreting or generalizing the result to determine the prevalence and seriousness of drug-induced arrhythmia and the association of each agent with the seriousness of arrhythmia. Moreover, although our studies identified medications with an uncommon, rare, or unknown risk of arrhythmia, medications such as anti-arrhythmic and sex-related hormones were not detected. In this regard, further large-scale clinical studies along with ongoing pharmacovigilance data collection are strongly warranted to elaborate on the clinical significance of drug-induced arrhythmia and to establish guidelines on safe use of medication with arrhythmic potentials in regards to age, sex, comorbidity, and concomitant medications, thereby promoting patient safety and improving patient outcomes.

## 4. Materials and Methods

### 4.1. Data Source and Definitions

This study was a cross-sectional study performed in accordance with the Strengthening the Reporting of Observational Studies in Epidemiology (STROBE) guideline to investigate the risk and prevalence of drug-induced arrhythmia utilizing KAERS DB, a nationwide spontaneous voluntary ADE reporting system, from January 2012 to December 2021 [50,51]. KAERS DB is available not only to the public but also to healthcare professionals, such as doctors, pharmacists, and nurses [51,52]. Also, ADE cases may be reported to the system by the local drug safety centers and drug manufacturers [51]. KAERS DB filters input errors and logical errors from the original data reported to the system, and all reported ADE cases are assessed for causalities, which are further verified by healthcare professionals appointed by the Korean Institute of Drug Safety and Risk Management (Ministry of Food and Drug Safety) based on patients’ medical records, scientific pharmacovigilance data from manufacturers, and interviews with patients and healthcare professionals to minimize potential biases [53]. All ADEs were reported in either include terms or preferred terms, according to the WHO-Adverse Reaction Terminology (WHO-ART) [54], which were then further classified into system organ class (SOC).

### 4.2. Data Acquisition

All ADE reports related to drug-induced arrhythmia from SOC classes, including cardiovascular disorders and heart rate and rhythm disorders, were collected from KAERS DB. The prespecified Medical Dictionary for Regulatory Activities (MedDRA) terminology related to drug-induced arrhythmia includes “arrythmia”, “atrial arrhythmia”, “cardiac arrythmia”, “arrhythmia sinus”, “ventricular arrythmia”, “dysrhythmias”, “bradyarrhythmia”, “supraventricular arrhythmia”, “sudden arrhythmic death syndrome”, “tachyarrhythmia”, and “torsade de pointes”, but is not limited to “long QT syndromes”, “electrocardiogram QT interval abnormal”, “electrocardiogram QT shortened”, “electrocardiogram QRS complex prolonged”, and “electrocardiogram QRS complex shortened”. Additionally, any ADEs designated as signs of arrhythmia, including palpitation, chest pain, and cardiac arrest or death secondary to arrhythmia by the Korean Institute of Drug Safety and Risk Management, were also included in the analysis. Only ADE cases with causality assessment results of “certain”, “probable/likely”, and “possible” per WHO-UMC were included in the analysis [54], and the following information was extracted from the system: (1) patient demographics, including age and sex; (2) medication information related to administration; (3) patient medical histories, including comorbidity and diagnosis for the medication uses; (4) causality assessment per WHO-UMC criteria such as “certain”, “probable/likely”, and “possible”; and (5) seriousness of reported ADEs based on the International Conference on Harmonization (ICH) E2D guideline [55]. Serious adverse events (SAEs) were classified as any AE resulting in (1) a life-threatening condition, (2) death, (3) hospitalization or prolongation of existing hospitalization, (4) congenital abnormalities or birth defects, (5) a persistent or significant disability or incapacity, and (6) other medically significant events identified in ICH E2D guidelines. The data extraction was performed in April 2023. This study protocol was approved by the Korean Institute of Drug Safety and Risk Management (Ministry of Food and Drug Safety) (No. 2212A0071) and the institutional review board (IRB) of Kyung Hee University (No. KHSIRB 23-123) (Seoul, Republic of Korea). Informed consents were exempted by the IRB.

### 4.3. Statistical Analysis: Descriptive Statistics

SPSS Statistics 28.0 (IBM SPSS Statistics for Windows, Armonk, NY, USA) and Rstudio (version 4.3.1) were utilized for all the statistical analysis. Descriptive statistics were conducted to analyze patient demographic information as well as ADE frequency. Continuous variables were expressed in median (range) according to the results of Kolmogorov–Smirnov normality testing. The risk of drug-induced arrhythmia for each medication was determined based on the frequency reported by Micromedex^®^ [56].

### 4.4. Statistical Analysis: Disproportionality and Predictor Identification Analysis

Disproportionality analysis was conducted to evaluate the association of medication classes and types with the seriousness of ADEs by estimating reporting odds ratios (RORs) with corresponding 95% confidence intervals (CIs) and Mantel–Haenszel adjusted *p*-values [57,58]. Medication classes with at least 5 reported cases for both SAEs and nonserious ADEs were subjected to the disproportionality analysis to ensure the reliability of the statistical analyses [58,59,60,61], and medications with at least 3 reported cases for both SAEs and nonserious ADEs were subjected to the disproportionality analysis due to the substantially low number of ADE cases for each medication. Additionally, associations between clinical predictors and the incidence of SAEs were evaluated by disproportionality analysis, and clinical predictors included age, sex, multiple medication therapy (at least 2 concomitant medications), presence of comorbidities, and history of cancer. Multiple logistic regression was carried out to determine the clinical impact of identified predictors on the potential risk of increasing SAEs, and the clinical predictors involve patient-specific characteristics such as age, sex, number of concomitant medications, and medical history. The enter method was applied to prevent a latent bias of selection and a covariate point of entry. The effect of each predictor was estimated with the OR and the corresponding 95% CI. Clinical plausibility was considered for the predictor analysis, and any *p*-values < 0.05 were considered statistically significant.

## 5. Conclusions

The foremost causative agent for drug-induced arrhythmia was sevoflurane, followed by paracetamol and pethidine, whereas fentanyl and pethidine were associated with the greatest number of serious arrhythmia cases. Medication classes involving antibiotics, chemotherapeutic agents, contrast media, and narcotic analgesics had a strong association with the seriousness of drug-induced arrhythmia. Among numerous etiologic agents, lidocaine, iodinated contrast media, and bisoprolol were more likely to be reported with serious drug-induced arrhythmia. Aging and the presence of a medical history, primarily cancer, are the major contributing factors for an increased risk of reporting SAEs. Nonetheless, further studies are warranted to elaborate on the prevalence and clinical significance of drug-induced arrhythmia and identify the risk factors. Meanwhile, simultaneous vigilant monitoring of the incidence of drug-induced arrhythmia is imperative to encourage patient safety.

## Figures and Tables

**Table 1:** Demographic information of ADE cases included in the analysis.

**Sex ^a^**	
Men	364 (55.0%)
Women	292 (44.1%)
**Age ^b^**	
<20	10 (1.5%)
20~39	29 (4.4%)
40~59	61 (9.2%)
60~79	197 (29.8%)
80~99	28 (4.2%)
**Causality**	
Certain	11 (1.7%)
Probable/Likely	91 (13.7%)
Possible	560 (84.5%)
**ADE type**	
Non-serious ADE	609 (92%)
Serious ADE	53 (8%)
Death	0 (0%)
Life-threatening condition	20 (37.7%)
Hospitalization and prolonged hospitalization	19 (35.8%)
Others	19 (35.9%)
**Reporter Types**	
Doctors	655 (98.9%)
Pharmacist	5 (0.8%)
Nurses	2 (0.3%)
**Indications/Comorbidities ^c^**	
Anesthesia	328 (49.5%)
Cancer	57 (8.6%)
Cardiovascular Disorders	55 (8.3%)
Infections	47 (7.1%)
Pain	11 (1.67%)
Psychiatric Disorders	4 (0.6%)
COVID-19	3 (0.5%)
Others	102 (15.4%)
**Number of Concomitant Medications**	
1	575 (86.9%)
2	60 (9.1%)
≥3	27 (4.1%)

^a^ missing in 6 (0.9%) cases ^b^ missing in 337 (50.1%) cases ^c^ missing in 55 (8.3%).

**Table 2:** Etiologic medications for drug-induced arrhythmia.

Drug Class	Risk of Arrhythmia ^a^	No SAE (*n* = 609)	SAE (*n* = 53)	Total (*n* = 662)
Anesthetic		336 (55.17%)	0 (0.00%)	336 (50.76%)
Sevoflurane	very rare	328 (53.86%)	0 (0.00%)	328 (49.55%)
Propofol	reported in overdosed patients (unknown)	8 (1.31%)	0 (0.00%)	8 (1.21%)
Antipyretic drugs		27 (4.43%)	0 (0.00%)	27 (4.08%)
Propacetamol Hydrochloride	unknown	22 (3.61%)	0 (0.00%)	22 (3.32%)
Acetaminophen	unknown	5 (0.82%)	0 (0.00%)	5 (0.76%)
Narcotic analgesic		35 (5.75%)	9 (16.98%)	44 (6.65%)
Pethidine Hydrochloride	reported as SAE (unknown)	15 (2.46%)	3 (5.66%)	18 (2.72%)
Fentanyl Citrate	reported as SAE (unknown)	1 (0.16%)	6 (11.32%)	7 (1.06%)
Tramadol Hydrochloride	common	11 (1.81%)	0 (0.00%)	11 (1.66%)
Morphine Hydrochloride	reported as SAE	7 (1.15%)	0 (0.00%)	7 (1.06%)
Oxycodone Hydrochloride	reported in overdosed patients	1 (0.16%)	0 (0.00%)	1 (0.15%)
Antihypertensive drugs		22 (3.61%)	4 (7.55%)	26 (3.93%)
Bisoprolol Fumarate	very common	8 (1.31%)	4 (7.55%)	12 (1.81%)
Nicardipine Hydrochloride	common	6 (0.99%)	0 (0.00%)	6 (0.91%)
Others	N/A	8 (1.31%)	0 (0.00%)	8 (1.21%)
Adrenal hormone drugs		21 (3.45%)	0 (0.00%)	21 (3.17%)
Norepinephrine Tartrate Hydrate	common	12 (1.97%)	0 (0.00%)	12 (1.81%)
Others	N/A	9 (1.48%)	0 (0.00%)	9 (1.36%)
Antibiotics		15 (2.46%)	5 (9.43%)	20 (3.02%)
Levofloxacin	common	5 (0.82%)	0 (0.00%)	5 (0.76%)
Cefazedone Sodium	unknown	0 (0.00%)	4 (7.55%)	4 (0.60%)
Ceftazidime Hydrate	unknown	4 (0.66%)	0 (0.00%)	4 (0.60%)
Others	N/A	6 (0.99%)	1 (1.89%)	7 (1.06%)
Peptic ulcer drugs		19 (3.12%)	0 (0.00%)	19 (2.87%)
Famotidine	unknown	18 (2.96%)	0 (0.00%)	18 (2.72%)
Rabeprazole Sodium	rare	1 (0.16%)	0 (0.00%)	1 (0.15%)
Antispasmodic drugs		15 (2.46%)	4 (7.55%)	19 (2.87%)
Cimetropium Bromide	reported in high doses (unknown)	11 (1.81%)	0 (0.00%)	11 (1.66%)
Atropine Sulfate Hydrate	common	0 (0.00%)	4 (7.55%)	4 (0.60%)
Scopolamine Butylbromide	uncommon	4 (0.66%)	0 (0.00%)	4 (0.60%)
Bronchodilator and Pulmonary acting agents		13 (2.13%)	0 (0.00%)	13 (1.96%)
Salbutamol Sulfate	rare	9 (1.48%)	0 (0.00%)	9 (1.36%)
Others	N/A	4 (0.66%)	0 (0.00%)	4 (0.60%)
Sympathomimetics		5 (0.82%)	0 (0.00%)	5 (0.76%)
Ephedrine Hydrochloride	rare	5 (0.82%)	0 (0.00%)	5 (0.76%)
Contrast media		11 (1.81%)	7 (13.21%)	18 (2.72%)
Iohexol	rare	4 (0.66%)	3 (5.66%)	7 (1.06%)
Ioversol	rare	3 (0.49%)	4 (7.55%)	7 (1.06%)
Others	N/A	4 (0.66%)	0 (0.00%)	4 (0.60%)
Psychotropic agent		10 (1.64%)	4 (7.55%)	14 (2.11%)
Quetiapine Fumarate	common	5 (0.82%)	0 (0.00%)	5 (0.76%)
Haloperidol	common	1 (0.16%)	1 (1.89%)	2 (0.30%)
Lorazepam	unknown	0 (0.00%)	2 (3.77%)	2 (0.30%)
Olanzapine	common with overdose	0 (0.00%)	1 (1.89%)	1 (0.15%)
Others	N/A	4 (0.66%)	0 (0.00%)	4 (0.60%)
Anti-malignant tumor drug		9 (1.48%)	5 (9.43%)	14 (2.11%)
Paclitaxel	uncommon	2 (0.33%)	3 (5.66)	5 (0.76%)
Oxaliplatin	common	4 (0.66%)	0 (0.00%)	4 (0.60%)
Fluorouracil	uncommon	2 (0.33%)	0 (0.00%)	2 (0.30%)
Trastuzumab	very common	2 (0.33%)	2 (3.77)	2 (0.30%)
Irinotecan Hydrochloride	unknown	1 (0.16%)	0 (0.00%)	1 (0.15%)
NSAIDs		13 (2.13%)	0 (0.00%)	13 (1.96%)
Nefopam Hydrochloride	unknown	4 (0.66%)	0 (0.00%)	4 (0.60%)
Ibuprofen	unknown	3 (0.49%)	0 (0.00%)	3 (0.45%)
Others	N/A	6 (0.99%)	0 (0.00%)	6 (0.90%)
Hypnotic sedative		7 (1.15%)	3 (5.66%)	10 (1.51%)
Dexmedetomidine Hydrochloride	rare	5 (0.82%)	2 (3.77%)	7 (1.05%)
Midazolam	uncommon	1 (0.16%)	1 (1.89%)	2 (0.30%)
Melatonin	unknown	1 (0.16%)	0 (0.00%)	1 (0.15%)
Local Anesthetic		4 (0.66%)	4 (7.55%)	8 (1.21%)
Lidocaine	unknown	4 (0.66%)	4 (7.55%)	8 (1.21%)
Muscle Relaxants		3 (0.49%)	3 (5.66%)	6 (0.90%)
Rocuronium	uncommon	3 (0.49%)	3 (5.66%)	6 (0.90%)
ETC	N/A	46 (7.55%)	3 (5.66%)	49 (7.40%)
Total	N/A	609 (100%)	53 (100%)	662 (100%)

^a^ determined based on the frequency of drug-induced arrhythmia. Classification: Very common (>1/10); common (>1/100, <1/10); uncommon (>1/1000, <1/100); rare (>1/10,000, <1/1000); very rare (<1/10,000); unknown. Abbreviations: N/A: not applicable; NSAIDs: non-steroidal anti-inflammatory drugs; SAE: serious adverse events.

**Table 3:** Predictors associated with increased risk of serious arrhythmia.

	ROR (95% CI)	*p-*Value	OR (95% CI)	*p-*Value
Age	5.153 (1.984–13.379)	<0.05	1.015 (0.9941–1.036)	>0.05
Sex (male)	1.480 (0.826–2.654)	0.239	1.640 (0.8250–3.296)	>0.05
Multiple Medications	1.193 (0.542–2.624)	0.821	0.568 (0.3074–1.049)	>0.05
Comorbidity (Patient History)	2.089 (1.000–4.367)	0.082	1.310 (0.4995–3.434)	>0.05
Cancer	2.779 (1.008–7.666)	0.040	0.600 (0.1064–3.380)	>0.05

ROR was estimated by disproportionality testing with Mantel–Haenszel-adjusted *p-*values, and OR was estimated by multiple logistic regression.

## Data Availability

Data are contained within the article.
